# Histopathologic growth patterns as prognostic factor for survival in patients with colorectal liver metastases

**DOI:** 10.2478/raon-2026-0002

**Published:** 2026-02-06

**Authors:** Sarkis Drejian, Mehdi Sadat Akhavi, Krzyztof Grzyb, Airazat Kazaryan, Åsmund Avdam Fretland, Bjørn Edwin, Davit Aghayan

**Affiliations:** The Intervention Centre, Oslo University Hospital, Oslo, Norway; Institute of Clinical Medicine, University of Oslo, Oslo, Norway; Department of Pathology, Oslo University Hospital, Oslo, Norway; Department of Surgery, Østfold Hospital Trust, Grålum, Norway; Department of Surgery N1, Yerevan State Medical University after M. Heratsi, Yerevan, Armenia; Department of Faculty Surgery N2, I. M. Sechenov First Moscow State Medical University, Moscow, Russia; Department of HPB Surgery, Oslo University Hospital, Oslo, Norway; Department of Surgery, Vestre Viken Hospital Trust, Ringerike Hospital, Hønefoss, Norway

**Keywords:** histopathologic growth pattern, survival, liver metastases, colorectal cancer

## Abstract

**Background:**

Histopathologic growth patterns (HGPs) of colorectal liver metastases (CRLM) have emerged as potential prognostic biomarkers, though their clinical significance remains under investigation. The objective is to evaluate the prognostic value of HGPs on recurrence-free survival (RFS) and overall survival (OS) in patients undergoing liver resection for CRLM.

**Patients and methods:**

This was a retrospective analysis of the OSLO-COMET randomized controlled trial, where 280 patients underwent laparoscopic or open parenchyma-sparing liver resection for CRLM between February 2012 and February 2016. Patients eligible for long-term analysis and with available histological material were included. HGPs were categorized as desmoplastic, pushing, replacement, or mixed, according to international consensus guidelines. Kaplan–Meier and Cox proportional hazards models were used to evaluate associations between HGPs and survival.

**Results:**

A total of 239 patients were included. Desmoplastic HGP was present in 43.5% of patients and associated with significantly better outcomes. Median RFS was 31 months for desmoplastic versus 9, 10, and 11 months for replacement, pushing, and mixed groups, respectively (p = 0.002). Five-year OS was 62% for desmoplastic, 59% for replacement, 55% for mixed, and 39% for pushing HGP (p = 0.036). In multivariable analysis, HGP, lymph node status, and extrahepatic disease were independent predictors of RFS. Age, tumor size, ECOG score, and extrahepatic metastasis significantly impacted OS.

**Conclusions:**

Replacement, pushing and mixed HGPs were associated with poor RFS, although replacement and mixed patterns showed better OS after treatment of recurrences. Desmoplastic HGP was independently associated with better RFS and OS following resection for CRLM.

## Introduction

Colorectal cancer ranks as the third most prevalent cancer globally. Nearly 50% of the patients diagnosed with colorectal cancer eventually develop liver metastases, contributing significantly to twothirds of all related lethal outcomes.^1^ Liver resection (LR), thermal ablation, chemotherapy (neoadjuvant and/or adjuvant), and liver transplantation for patients with colorectal liver metastases (CRLM) are potential curative treatment methods. In order to choose the best treatment methods and a personalized approach for each patient, different clinical, histopathological, and genomic factors are being studied continuously.^2–6^

Today, established scoring systems like the Fong clinical risk score (CRS)^7^, Basingstoke predictive index (BPI)^8^, RAS mutation clinical score, and Tumor burden score are widely used to predict outcomes in CRLM patients.^9,10^ These scoring systems use variables such as tumor size, number of tumors, disease-free interval, carcinoembryonic antigen (CEA) level, positive resection margin, presence of extrahepatic disease, positive lymph nodes in the primary tumor, and tumor mutation status as critical predictors of recurrence-free and overall survival.

**FIGURE 1.**
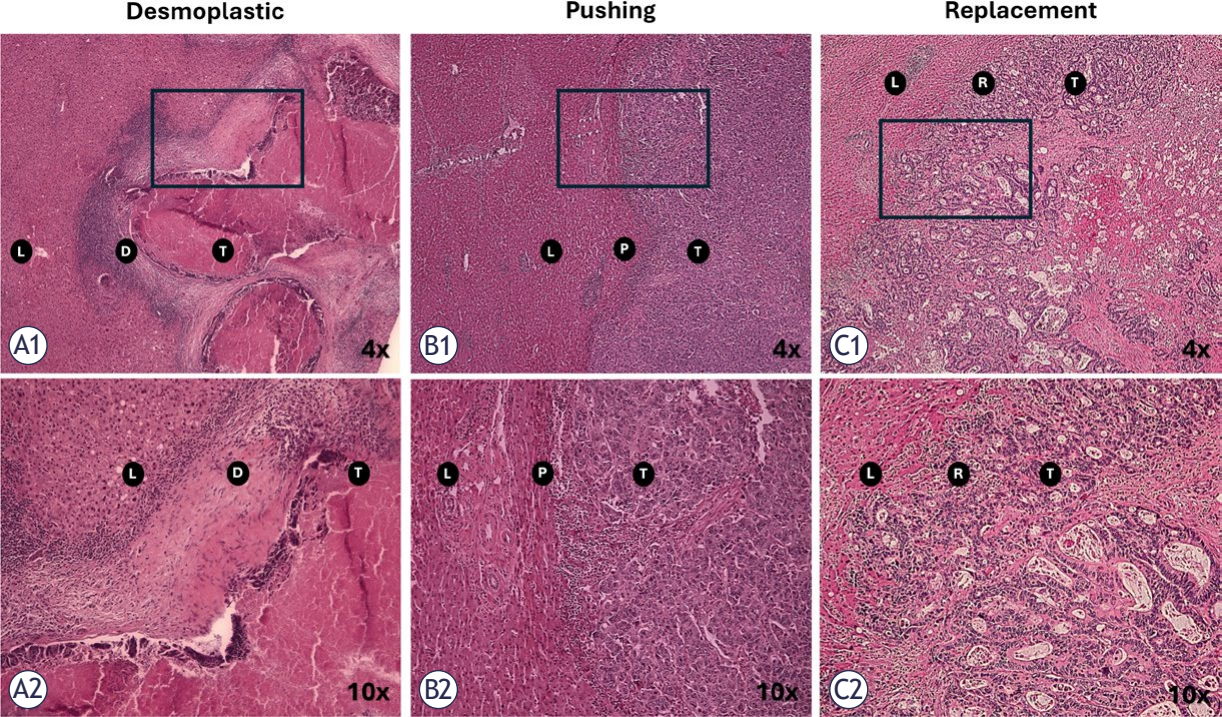
Haematoxylin and eosin (H & E) evaluation of the three growth patterns of colorectal liver metastasis. **(A1)** Desmoplastic pattern (fibrotic tissue as a barrier between tumor and liver parenchyma), 4x, **(A2)** Desmoplastic pattern, 10x, **(B1)** Pushing pattern (hepatocytes compressed by tumor), 4x, **(B2)** Pushing pattern, 10x, **(C1)** Replacement pattern (liver parenchyma is infiltrated by tumor cells), 4x, **(C2)** Replacement pattern, 10x. The photos are from OSLO-COMET RCT archive. D = Desmoplastic; L = Liver; P = Pushing; R = Replacement; RCT = randomized controlled trial; T = tumor

In 2001, Vermeulen *et al*. described three histopathological growth patterns (HGPs) according to the interaction between CRLM and surrounding liver tissue: *desmoplastic* – tumor is surrounded by fibrotic tissue, pushing – tumor is compressing the adjacent hepatocytes, and *replacement* – when there is tumor infiltration into the liver parenchyma (Figure 1).^11^ Van Dam *et al*., in 2017 advocated reproducibility in the determination of growth patterns for CRLM through an international consensus guideline.^12^ In 2022, Latacz *et al*. published an updated international consensus guideline for scoring growth patterns, where they assessed the HGPs by categorizing them into two groups – pure desmoplastic and non-desmoplastic.^13^

Numerous studies examined the impact of the HGPs on survival and recurrence. The majority of these studies demonstrated that desmoplastic HGP is associated with more favorable outcomes.^14–16^ However, there is no unanimity regarding replacement and pushing HGPs. Some studies concluded that pushing HGP is a predictor for poor overall survival^14^, while others identified replacement HGP as an independent predictor of poor survival.^15^ Additionally, Torén *et al*. have found no significant impact of HGPs on overall survival and recurrence.^17^

Despite ongoing debate and variability in reported findings, investigation of HGPs remains highly relevant, particularly in light of the increasing interest in incorporating HGP assessment into the standardized histopathological evaluation of CRLM.^18^

This study aims to examine the impact of the HGPs on patient survival and their value as an individual prognosticating factor in patients undergoing liver resection for CRLM.

## Patients and methods

### Clinical data

OSLO-COMET (ClinicalTrials.gov: NCT01516710) was a single-centre, randomized controlled trial that recruited patients from Oslo University Hospital in Oslo, Norway. It was approved by the Regional Ethical Committee of South Eastern Norway (REK Sør-Øst B 2011/1285) and the data protection officer of the Oslo University Hospital. Between February 2012 to February 2016, 280 patients with colorectal liver metastases were included in the study for liver resection.^2,19^

**Figure 2.**
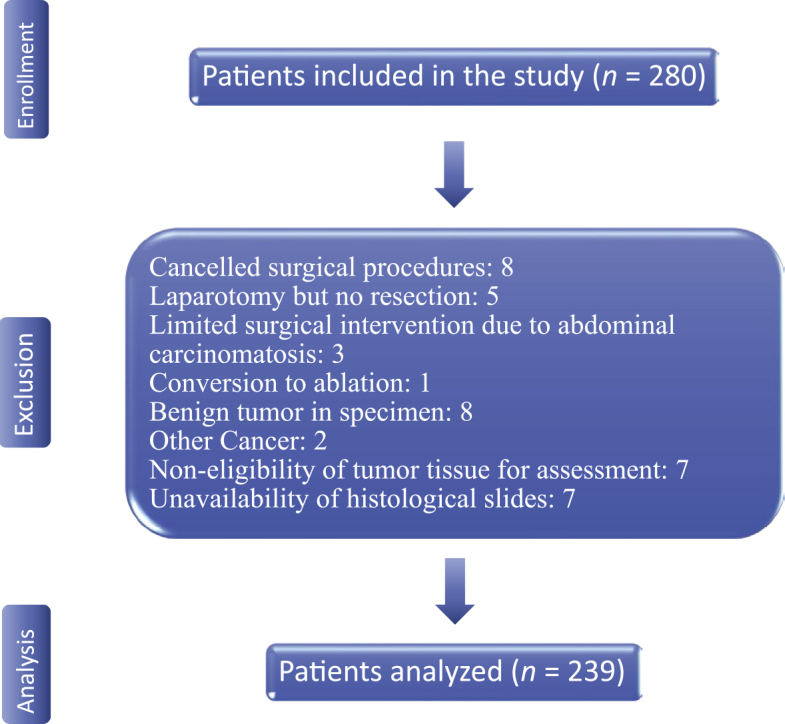
Study flow diagram.

A total of 239 patients were identified from the pathology archives of the OSLO-COMET trial by the first two authors. Clinical data were retrieved from the trial’s prospectively maintained database. Forty-one patients were excluded for the following reasons: Non-eligibility of tissue material for histopathological growth pattern assessment, unavailability of histological slides, cancelled surgical procedures, conversion to ablation, surgical intervention limited to biopsy due to the presence of abdominal carcinomatosis, diagnosis of benign and other tumors (Figure 2).

### Histopathologic growth patterns (HGP)

HGPs were evaluated at the Department of Pathology, Oslo University Hospital, by the two co-authors in collaboration with an expert pathologist. The assessment followed standardized international consensus criteria, with the classification method described by Nielsen *et al*. serving as a reference.^15^ For each case, all available hematoxylinand-eosin–stained sections were reviewed microscopically, and findings were discussed until consensus was reached. Tumors in which more than 50% of the tumor–liver interface displayed a single growth pattern were classified according to that pattern, whereas those with two or more patterns present in equal proportions were categorized as mixed growth pattern.

In total, 425 tumors from 239 patients were analyzed. Among these, 95 patients presented with multiple tumors, accounting for 281 tumors in total. Of the patients with multiple tumors, 31 had tumors with different HGPs. In such cases, the patient’s overall growth pattern classification assigned according to the HGP with the poorest prognosis.

### Statistical analysis

Descriptive data are presented with medians, interquartile ranges, numbers, and percentages. Categorical variables were compared using the chi-square or Fisher’s exact test when applicable. Continuous variables were compared using Kruskal-Wallis and Anova test for non-normally and normally distributed variables, respectively. Post-hoc test was applied when relevant.

Overall survival (OS) was estimated from liver resection until death, and recurrence-free survival (RFS) was estimated from liver resection until the first radiologic proof of disease recurrence. Survival data for the treatment groups were analyzed with Kaplan-Meier method and, log-rank test were applied to test the equality of survival curves.

To identify predictors of RFS and OS, both univariable and multivariable analyses were conducted using the Cox proportional hazards model. All variables associated with survival that had p-values ≤ 0.2 in the univariable analysis were subsequently included in a Cox multivariable regression model^20^, and p-values ≤ 0.05 were considered statistically significant.

## Results

### Baseline characteristics and growth pattern analysis

The prevalence of the HGPs in the study cohort was desmoplastic in 104 patients (43.5%), pushing in 54 (22.6%), replacement in 32 (13.3%), and mixed in 49 (20.5%). The clinical characteristics were largely balanced across the HGP groups, except for the age, where the median age was higher in the group with pushing growth pattern (p-value = 0.028), and the tumor size, where pushing and mixed growth patterns had larger tumors (p-value = 0.003) (Table 1).

**Table 1.** Baseline characteristics

Variable	Total n = 239	Desmoplastic HGP n = 104	Pushing HGP n = 54	Replacement HGP n = 32	Mixed HGP n = 49	P-value
Male	141 (59%)	68 (65%)	34 (62%)	14 (44%)	25 (51%)	0.091
Median age (IQR), years^*^	67 (60–74)	66 (58–73)	70 (63–76)	62.5 (58–71)	68 (64–73)	0.028
Median BMI (IQR), kg/m^2^	24.8 (22.53–28.03)	24.89 (22.85–28.6)	24.68 (23.0–26.56)	24.93 (21.71–27.68)	24.8 (21.85–28.41)	0.585
ECOG score						
0	197 (82%)	85 (82%)	46 (85%)	26 (81%)	40 (82%)	
1	40 (17%)	18 (17%)	8 (15%)	5 (16%)	9 (18%)	0.843
2	2 (1%)	1 (1%)	0	1 (3%)	0	
ASA score						
1	27 (11%)	13 (13%)	6 (11%)	7 (22%)	2 (4%)	
2	120 (50.5%)	49 (47%)	25 (46%)	16 (50%)	30 (61%)	0.151
3	90 (38%)	42 (40%)	22 (41%)	9 (28%)	17 (35%)	
4	1 (0.5%)	0	1 (2%)	0	0	
Primary tumor in the rectum	99 (41%)	44 (42%)	22 (41%)	14 (44%)	19 (39%)	0.968
Primary tumor lymph node metastasis	158 (66%)	5 (57%)	41 (76%)	26 (81%)	32 (65%)	0.021
Primary tumor						
AJCC stage						
T1	5 (2%)	3 (3%)	0	0	2 (4%)	
T2	14 (6%)	2 (2%)	5 (9%)	2 (6%)	5 (10%)	0.116
T3	164 (69%)	79 (76%)	31 (58%)	21 (66%)	33 (67%)	
T4	56 (23%)	20 (19%)	18 (33%)	9 (28%)	9 (19%)	
Synchronous	140 (59%)	56 (54%)	34 (63%)	18 (56%)	32 (65%)	0.499
Chemotherapy within 6 months prior to surgery	121 (51%)	55 (53%)	27 (50%)	17 (53%)	22 (45%)	0.815
Median CEA level (IQR), μg/L	4.3 (2.35–9.5)	4.0 (2.45–8.4)	3.9 (1.8–10.5)	5.65 (2.47–6.25)	5.0 (2.6–8.05)	0.731
Previous liver resection	29 (12%)	13 (13%)	6 (11%)	5 (16%)	5 (10%)	0.896
Median tumor size (IQR), cm	2.4 (1.50–3.50)	2.1 (1.30–3.40)	2.5 (2.08–4.08)	1.8 (1.40–3.15)	2.5 (1.95–4.00)	0.003
Minimum/maximum	0.6–16	0.7–9	0.6–8	0.8–7.5	0.8–16	
Open liver resection	126 (53%)	55 (53%)	28 (52%)	17 (53%)	26 (53%)	1.000
Multiple lesions	95 (40%)	37 (36%)	26 (48%)	10 (31%)	22 (45%)	0.278
Right colon	39 (16%)	15 (14%)	10 (19%)	4 (13%)	10 (20%)	0.704

1 AJCC = American Joint Committee on Cancer; ASA = American Society of Anesthesiologists; BMI = body mass index; CEA = carcinoembryonic antigen; ECOG = Eastern Cooperative Oncology Group; HGP = histopathologic growth pattern; IQR = interquartile range.

* Post-hoc test for variables with significant differences

1 Age: Desmoplastic *vs*. Pushing (p-value 0.036), Pushing *vs*. Replacement (p-value 0.010), Replacement *vs*. Mixed (p-value 0.032).

1 Primary tumor lymph node metastasis: Desmoplastic *vs*. Pushing (p-value 0.018), Desmoplastic *vs*. Replacement (p-value 0.012)

1 Median tumor size: Desmoplastic *vs*. Pushing (p-value 0.04), Desmoplastic *vs*. Mixed (p-value 0.020), Pushing *vs*. Replacement (p-value 0.007), Replaceme*nt vs. Mixed (p-value 0.022)*

### Long-term oncologic outcomes

**Figure 3.**
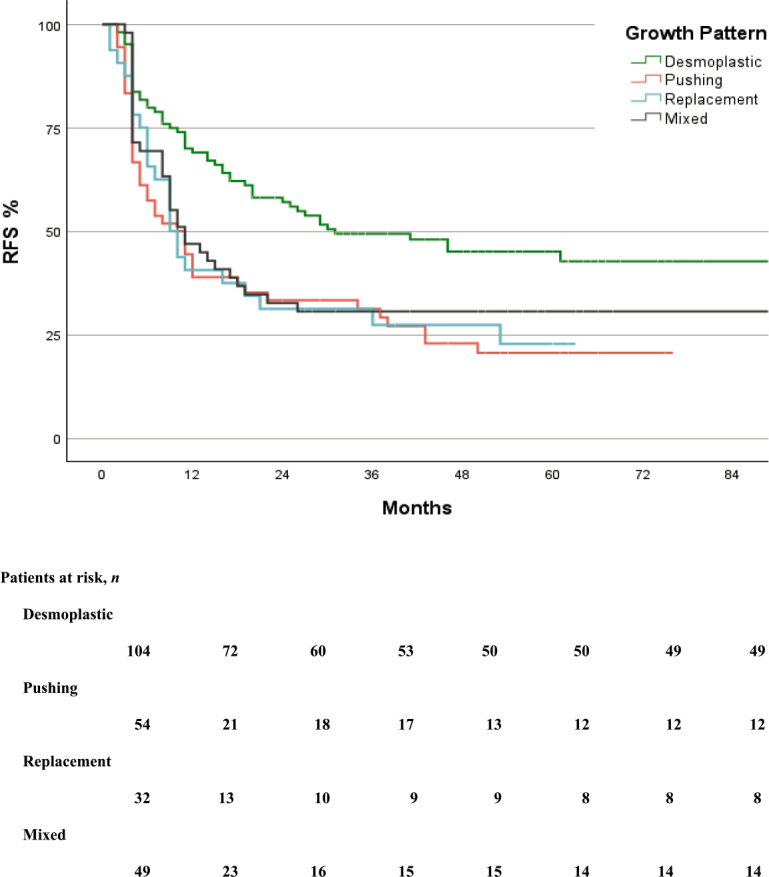
Kaplan-Meier curve of recurrence-free survival in the groups.

**Figure 4.**
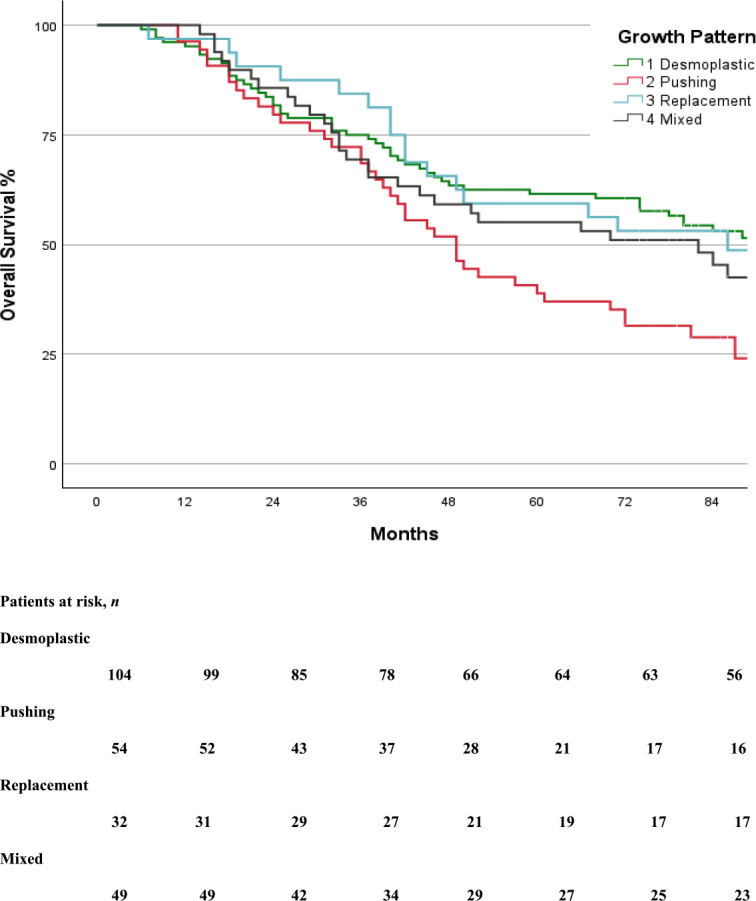
Kaplan-Meier curve of overall survival in the groups.

In total, 155 (65%) patients had disease recurrence. RFS was significantly better in the group with desmoplastic growth pattern (p-value = 0.006) compared to non-desmoplastic. The median RFS for total cohort was 16 months (95% CI, 11 to 21 months), (p-value = 0.002) (Figure 3) whereas, the median OS was 74 months (95% CI, 58 to 90 months), (p-value = 0.036) (Table 2, Figure 4).

**Table 2.** Chemotherapy administration and long-term oncologic outcomes

Variable	All types 239	Desmoplastic 104	Pushing 54	Replacement 32	Mixed 49	P-value
**Neoadjuvant chemotherapy, n (%)**	95 (40%)	46 (44%)	19 (35%)	12 (38%)	18 (37%)	0.660
**Adjuvant chemotherapy, n (%)**	122 (51%)	53 (51%)	25 (46%)	12 (38%)	32 (65%)	0.078
**Recurrence-free survival**						
1 y, %	54%	69%	39%	41%	47%	
3y, %	38%	49%	31%	27%	31%	
5y, %	33%	45%	21%	23%	31%	
Median (95% CI), mo	16 (11 to 21)	31 (12 to 50)	10 (5 to 15)	9 (6 to 12)	11 (6 to 16)	**0.002**
**Disease recurrence, n (%)**	155 (65%)	55 (53%)	42 (78%)	24 (75%)	34 (70%)	**0.006**
Liver, n	84 (35%)	33 (32%)	22 (42%)	9 (29%)	20 (41%)	0.415
Isolated liver recurrence	58 (24%)	24 (23%)	15 (28%)	6 (19%)	13 (27%)	0.750
Recurrence in resection bed	9 (4%)	5 (5%)	3 (6%)	1 (3%)	0	0.433
Lung, n	60 (25%)	18 (17%)	17 (31%)	12 (38%)	13 (27%)	0.066
Other Extrahepatic, n	37 (16%)	15 (14%)	9 (17%)	5 (16%)	8 (16%)	0.982
**Treatment of the first disease recurrence**						
Repeated liver surgery, n (%)	56 (23%)	20 (19%)	16 (30%)	7 (22%)	13 (27%)	0.500
LLR	29 (12%)	13 (13%)	5 (9%)	3 (9%)	8 (16%)	
OLR	20 (8%)	7 (7%)	6 (11%)	2 (6%)	5 (10%)	
RFA	6 (3%)	0	5 (9%)	1 (3%)	0	
Transplant	1 (0.5%)	0	0	1 (3%)	0	
Lung resection, n (%)	13 (5%)	4 (4%)	1 (2%)	6 (20%)	2 (4%)	**0.005**
Other surgical procedure, n (%)	9 (4%)	5 (5%)	2 (4%)	2 (6%)	0	0.428
Only palliative chemotherapy, n (%)	43 (18%)	13 (13%)	13 (24%)	5 (16%)	12 (25%)	0.172
Radiotherapy and radio-chemotherapy, n (%)	24 (10%)	9 (9%)	7 (13%)	4 (13%)	4 (8%)	0.768
No treatment, n (%)	10 (4%)	5 (5%)	4 (7%)	1 (3%)	0	0.292
**Overall survival**						
1 y, %	97%	95%	96%	97%	98%	
3 y, %	74%	75%	72%	84%	69%	
5 y, %	55%	62%	39%	59%	55%	
Median (95% CI), months	74 (58 to 90)	94	49 (39 to 59)	86 (43 to 129)	82 (43 to 121)	**0.036**
Alive patients, n (%)	103 (43%)	52 (50%)	16 (30%)	14 (44%)	22 (45%)	0.064

1 HR = hazard ratio; IQR = interquartile range; CI = confidence interval; n= number; mo = month; LLR = laparoscopic liver resection; OLR = open liver resection; RFA = radiofrequency ablation; Y = year

* Post-hoc test for variables with significant differences –

1 Recurrence-free survival: Desmoplastic *vs*. Pushing (p-value < 0.001), Desmoplastic *vs*. Replacement (p-Value 0.007),

1 Desmoplastic *vs*. Mixed (p-Value 0.023)

1 Disease recurrence: Desmoplastic *vs*. Pushing (p-value 0.002), Desmoplastic *vs*. Replacement (p-value 0.027)

1 Lung resection: Desmoplastic *vs*. Replacement (p-value 0.05), Pushing *vs*. Replacement (p-value 0.006), Replacement *vs*. Mixed (p-value 0.031)

1 Overall survival: Desmoplastic *vs*. Pushing (p-value 0.005), Pushing *vs*. Replacement (p-value 0.049

### Prognostic factors

In multivariable Cox regression analyses, lymph node status of primary tumor, extrahepatic metastasis and HGPs were independent predictors of recurrence-free survival, while age, ECOG score, tumor size and extrahepatic metastasis were independent predictors for overall survival (Table 3).

**Table 3.** Univariable and multivariable analysis of prognostic factors for recurrence free and overall survival

Variable	Recurrence-free survival			Overall survival		
	Univariable	Multivariable Cox regression analysis		Univariable	Multivariable Cox regression analysis	
	P Value	Hazard ratio (95% CI)	P-Value	P-value	Hazard ratio (95% CI)	P-value
Age (per year)	0.463			**< 0.001**	**1.027 (1.007–1.047)**	**0.008**
Male sex	0.579			**0.193**	**1.432 (0.993–2.065)**	0.055
BMI	**0.126**	**1.029 (0.989–1.070)**	0.156	0.697		
ECOG score	**0.130**	1.273 (0.868– .869)	0.217	**0.005**	**1.659 (1.129–2.437)**	**0.010**
ASA score	**0.108**	**1.119 (0.858–1.458)**	0.408	**0.192**		
***Primary tumor***						
Rectum	**0.195**	**0.885 (0.624–1.256)**	0.494	0.403		
Right colon	0.75			**0.134**	**1.238 (0.798–1.919)**	0.341
AJCC T- stage (1,2–3,4)	**0.176**	**0.547 (0.291–1.029)**	0.061	0.597		
Lymph node involvement	**< 0.001**	**1.610 (1.075–2.412)**	**0.021**	**0.041**	1.349 (0.921–1.976)	0.124
***Liver metastasis***						
Synchronous	**0.023**	1.302 (0.914–1.856)	0.144	**0.129**		
Previous liver resection	0.791			0.256		
Multiple lesions	0.310			0.798		
Lobar distribution	0.621			0.494		
Tumor size (per cm)	**0.017**	**1.084 (0.992–1.185)**	0.076	**< 0.001**	**1.014 (1.005–1.022)**	**0.002**
Chemo within 6 mo prior surgery	**0.109**	**1.140 (0.814–1.597)**	0.446	0.997		
Preoperative CEA (> 5 ng/mL)	**0.155**	**1.255 (0.896–1.757)**	0.186	0.750		
Extrahepatic disease	**< 0.001**	**2.834 (1.766–4.548)**	**< 0.001**	**0.011**	**1.690 (1.028–2.779)**	**0.039**
***Liver resection***						
Laparoscopy	0.489			0.515		
Blood loss	0.486			0.863		
Blood transfusion	0.353			0.243		
Operative time	0.896			0.684		
Post operative severe complications	0.900			0.216		
R1 resection (<1 mm)^*^	**0.094**	**0.831 (0.504–1.370)**	0.468	**0.120**	**1.075 (0.663–1.741)**	0.77
Involved resection margin	**0.002**	**1.625 (0.841–3.141)**	0.148	**0.156**	**1.390 (0.730–2.648)**	0.316
No adjuvant chemotherapy	0.401			0.555		
***Growth patterns***						
Desmoplastic *vs*. none desmoplastic	**< 0.001**	**1.446 (1.005–2.081)**	**0.047**	**0.073**	**0.912 (0.633–1.313)**	0.619

1 AJCC = American Joint Committee on Cancer; ASA = American Society of Anesthesiologists; BMI = body mass index; CEA = carcinoembryonic antigen; ECOG = Eastern Cooperative Oncology Group; mo = month

* R1 resection - presence of tumor cells within 1 mm from the resection margin

### Discussion

In this follow-up study of OSLO-COMET, we here report a significant correlation between HGP and long-term oncological outcomes in patients operated for colorectal cancer liver metastases. The desmoplastic growth pattern demonstrated a better RFS compared to pushing, replacement, and mixed growth patterns. Replacement and mixed growth patterns were associated with poorer RFS, but better OS, possibly indicating a phenotype that responds well to treatment of recurrence. The pushing growth pattern was correlated to the poorest OS.

Notably, while the HGPs were established as independent predictors for RFS in multivariable analyses, they did not remain significant predictors in the final multivariable Cox regression analysis for OS. This finding may be partly attributed to collinearity between HGP and tumor size, a variable that retained significance in our analysis. This finding contrasts with the study conducted by Nielsen *et al*.^15^, which identified both HGP and tumor size as independent predictors of OS. A significant disparity in tumor size distribution was observed across HGP subgroups; patients with pushing and mixed growth patterns presented with larger tumors compared to those exhibiting desmoplastic and replacement patterns—similar to the findings of Van den Eynden *et al*.^14^ Since larger tumors tend to correlate with adverse outcomes^21,22^, this imbalance may have confounded the relationship between HGP and survival, diminishing the independent prognostic role of histopathologic growth patterns when adjusted for tumor size, subsequently leading to its exclusion from the final analysis.

In terms of disease recurrence, desmoplastic HGP was associated with a lower recurrence rate (53%) compared to pushing (78%) and replacement (75%), with mixed growth patterns showing a recurrence rate of 70% (p-value = 0.006). The increased recurrence rates observed in non-desmoplastic HGPs may reflect underlying biological characteristics and differences. The tumors with desmoplastic HGP are characterized by a fibrotic stromal rim that separates tumor cells from adjacent hepatocytes and is frequently associated with a pronounced immune cell infiltration.^23^ This tumor-host interface may act as both a mechanical and immunologic barrier, which could account for the improved RFS seen in desmoplastic patients. In contrast, non-desmoplastic growth patterns have minimal inflammatory response, potentially facilitating higher risk of recurrence despite complete resection, and suggesting more aggressive biological behaviour. Furthermore, the likelihood of achieving curative-intent treatment upon recurrence differed among growth pattern groups, with lung recurrence being notably more frequent in the replacement group (38%) than in the pushing group (31%), and lower in mixed (27%) and desmoplastic groups (17%). Notably, lung resections were performed more frequently in the replacement group (20%, p-value = 0.005), possibly indicative of a distinct metastatic growth pattern. Conversely, the rates of repeat liver surgery did not significantly differ across groups (p-value = 0.500), suggesting that growth patterns did not preclude surgical intervention for liver recurrences. The patients with replacement and mixed HGPs, despite their poorer RFS, appeared to derive benefit from treatment at recurrence, as supported by the clear improvement in survival curves shown in Figures 3 and 4. This pattern was not observed in patients with pushing HGP; although these patients received similar treatment strategies for recurrence, their OS curve did not demonstrate a significant benefit, indicating a need for further, more detailed investigation.

An intriguing observation within our cohort was the older median age of patients with pushing growth patterns (70 years) in comparison to other HGPs, while the replacement pattern was more prevalent among younger patients (median age 62.5). This age-related distribution may reflect intrinsic differences in tumor biology or variations in the host immune response. Furthermore, tumor size was markedly larger in the pushing and mixed groups (median 2.5 cm) compared to the replacement group (median 1.8 cm), suggesting a more expansive growth behaviour in these patterns. Lymph node metastasis was significantly more prevalent among patients with pushing (76%) and replacement (81%) growth patterns, implying a correlation with a more aggressive tumor biology and heightened metastatic potential.^24,25^ These findings are consistent with those reported by Galjart *et al*., who underscored the importance of vascularization in HGP classification.^16^ Specifically, the replacement pattern, characterized by vessel cooption rather than angiogenesis, may influence differences in tumor dissemination and immune evasion^26^, which may be possible explanation for both frequent recurrences and high percentage of lymph node prevalence in this group.

In comparing our findings to previous research, Nielsen *et al*. concluded that the replacement HGP had the most adverse effect on survival outcomes. This discrepancy may stem from differences in the variables incorporated into the Cox model. Our results are more consistent with those presented by Van den Eynden *et al*.^14^, who identified the worst OS in patients with pushing HGP, and with Galjart *et al*.^16^, though the latter did not distinctly categorize pushing, replacement, and mixed subtypes. Conversely, Torén *et al*. found no significant correlation between HGP and survival outcomes, which may be attributed to several factors, including the limited sample size of pushing HGP (25 patients) and the absence of mixed subtypes.^17^

Overall, our findings suggest that desmoplastic HGPs are independently associated with better oncological outcomes, even after adjusting for established clinicopathologic variables and treatment strategies. This can be a supportive notion that HGPs may serve as surrogate biomarkers for tumor biology, and their integration into clinical decision-making has the potential to enhance surveillance and therapeutic strategies following resection or ablation.

The study has several limitations that should be recognized when interpreting the results. The sample size is relatively modest, particularly when stratified into the four HGP subgroups. Although our sample size is comparable to the majority of studies in this domain, future research should aim to include larger datasets for more robust conclusions. Additionally, the limited number of cases in certain subgroups, such as replacement and pushing patterns, may reduce statistical power.

Although histopathological evaluations of growth patterns were carried out in accordance with established international guidelines and under expert supervision, the potential for selection bias cannot be dismissed. Instances of considerable tumor size (e.g., 5 cm in diameter) were represented by only a handful of slides, which may lead to underrepresentation of specific growth patterns. Consequently, critical histological features may have been overlooked, resulting in misclassification of some tumors concerning their dominant HGP. Moreover, the microscopic evaluation and classification remain inherently subjective and susceptible to interobserver variability, despite the specialists’ expertise; inconsistencies in interpretation between institutions remain an ongoing challenge in the accurate assessment of HGPs.

To address some of these limitations, we plan to employ artificial intelligence (AI)-based algorithms for histopathological evaluation of HGPs in the future, aiming for enhanced accuracy.^27^ AI may provide a standardized, quantitative analysis of the tumor-liver interface, by mitigating human error and bias while offering more reproducible and precise assessments. Integrating AI tools could strengthen the reliability of growth pattern classification and enhance its utility as a prognostic marker in survival analyses.

In contemporary medicine, the focus of disease treatment is shifting towards personalized approaches that are tailored to individual patient characteristics rather than solely emphasizing the disease itself.^28^ Consequently, researchers are increasingly investigating clinical features that may play a significant role in personalized treatment strategies. In this study, we aimed to assess the importance of HGPs in predicting RFS and OS, an area that remains relatively novel and underexplored compared to more well-characterized clinical and pathological prognostic factors. ^7–9,29^ A deeper understanding of HGPs may facilitate treatment opportunities for patients previously denied surgical options due to unfavorable clinical and pathological prognostic characteristics.

## Conclusions

In conclusion, replacement, pushing, and mixed growth patterns were associated with poorer RFS, with the pushing growth pattern was also associated with poor OS. The replacement and mixed growth patterns demonstrated improved OS following the treatment of recurrences. The desmoplastic growth pattern was associated with superior RFS and OS compared to other groups. Incorporating HGPs into routine histopathology reports may enhance risk stratification and guide postoperative surveillance strategies. Future studies with larger, multicenter datasets are warranted to validate these findings. Overall, HGPs hold promise for enhancing personalized treatment planning in the management of colorectal liver metastases.
